# Brigatinib, an anaplastic lymphoma kinase inhibitor, abrogates activity and growth in ALK-positive neuroblastoma cells, *Drosophila* and mice

**DOI:** 10.18632/oncotarget.8508

**Published:** 2016-03-31

**Authors:** Joachim T. Siaw, Haiying Wan, Kathrin Pfeifer, Victor M. Rivera, Jikui Guan, Ruth H. Palmer, Bengt Hallberg

**Affiliations:** ^1^ Department of Medical Biochemistry and Cell Biology, Institute of Biomedicine, Sahlgrenska Academy, University of Gothenburg, Gothenburg, Sweden; ^2^ ARIAD Pharmaceuticals, Inc., Cambridge, Massachusetts, USA

**Keywords:** anaplastic lymphoma kinase, neuroblastoma, brigatinib, AP26113, resistant mutations

## Abstract

Anaplastic lymphoma kinase (ALK) is a tyrosine kinase receptor which has been implicated in numerous solid and hematologic cancers. ALK mutations are reported in about 5-7% of neuroblastoma cases but the ALK-positive percentage increases significantly in the relapsed patient population. Crizotinib, the first clinically approved ALK inhibitor for the treatment of ALK-positive lung cancer has had less dramatic responses in neuroblastoma. Here we investigate the efficacy of a second-generation ALK inhibitor, brigatinib, in a neuroblastoma setting. Employing neuroblastoma cell lines, mouse xenograft and *Drosophila melanogaster* model systems expressing different constitutively active ALK variants, we show clear and efficient inhibition of ALK activity by brigatinib. Similar abrogation of ALK activity was observed *in vitro* employing a set of different constitutively active ALK variants in biochemical assays. These results suggest that brigatinib is an effective inhibitor of ALK kinase activity in ALK addicted neuroblastoma that should be considered as a potential future therapeutic option for ALK-positive neuroblastoma patients alone or in combination with other treatments.

## INTRODUCTION

The development of pharmacologic strategies targeting anaplastic lymphoma kinase (ALK) reflects the increasing involvement of ALK in a subset of human malignancies where ALK is well accepted as an initiator and progression marker, representing a tractable oncogene for targeted therapy. The original discovery of ALK was in 1994, when Morris and colleagues first characterized the *ALK* gene as a fusion partner of nucleophosmin (NPM), in the *NPM-ALK* translocation found in a subset of anaplastic large cell lymphoma (ALCL) [1]. Further studies have revealed numerous different ALK fusion proteins in other tumors such as inflammatory myofibroblastic tumor (IMT), diffuse large B cell lymphoma (DLBCL) and non-small cell lung cancer (NSCLC) among others [2, 3].

First and second generation ALK inhibitors, such as crizotinib and ceritinib, which have been FDA approved [4], provide hope for a targeted therapy in patients with aberrant ALK activity. An additional ALK inhibitor, alectinib (CH5424802) has been approved in Japan for use in ALK-positive NSCLC [5]. While the above mentioned drugs are all ATP-competitive inhibitors of ALK, they differ in their binding properties and display differential activity in blocking the activity of the various ALK resistant mutant forms [3, 6, 7]. Thus, a complex picture of ALK inhibition is emerging, with an increasing number of reports suggesting distinct patterns of resistance mutations arising following primary treatment with particular ALK inhibitors.

The situation in pediatric neuroblastoma is further complicated by the fact that point mutations in ALK occur as primary, and potentially driver mutations in therapy naïve patients. Neuroblastoma, a tumor of the developing nervous system accounts for 15% of all pediatric oncology death [8, 9]. Neuroblastoma is a heterogeneous disease and while a subset may undergo spontaneous differentiation or regression with little or no therapy, the majority are difficult to cure with current regimes [8, 9]. The most common genetic features of neuroblastoma are amplification of the proto-oncogene *MYCN*, deletions of parts of chromosome arms 1p and 11q, gain of parts of 17q and triploidy [8–10], and mutations in the kinase domain of ALK which occur in both sporadic and familial forms of neuroblastoma [11–15]. A recent trial of crizotinib in ALK-positive pediatric cancers demonstrated excellent activity in pediatric ALCL, where the NPM-ALK fusion protein is the major driver, but suboptimal activity in neuroblastoma [16]. At the present time it is not clear whether this reflects suboptimal inhibitory properties or possibly reflects the heterogeneous nature of neuroblastoma in terms of chromosomal irregularities, since neuroblastoma exhibits both numerical and segmental chromosome alterations [8, 17–20]. ALK aberrations in neuroblastoma are predominantly point mutations in the context of full-length ALK however other variants, such as deletions have also been reported [21, 22]. ALK mutation occurs in about 5-7% of neuroblastoma cases but this percentage is increased significantly in the relapsed patient population where approximately 20-25% of patients have ALK mutations [23]. This has been verified in an additional study of relapsed tumors in which the percentage of ALK-positive relapsed patients was reported as greater than 40% [24, 25]. It has been reported that neuroblastoma patients bearing both *MYCN* amplification and ALK mutations are characterized by unfavorable aggressive neuroblastoma phenotype [26]. Activating ligands for ALK have recently been identified as FAM150A and FAM150B [27, 28]. These small secreted ligands are able to drive ‘super activation’ of activated ALK mutants from neuroblastoma suggesting dysregulation of the ALK ligands may play a role in neuroblastoma [27]. Further characterization of the FAM150 mediated ligand activation of ALK signaling should clarify the significance of the ligand-ALK interaction as a potential therapeutic target. Thus, in the context of neuroblastoma, a number of approaches are actively being explored for therapeutic intervention, with evaluation of new ALK inhibitors a high priority.

Brigatinib, also known as AP26113, is one of the most recently described second generation ALK inhibitors [6]. Clinical trial data reports that about 72% of crizotinib refractory ALK-positive NSCLC patients responded to treatment with brigatinib [29]. Based on these encouraging clinical responses in NSCLC, we decided to explore the therapeutic potential of brigatinib in the context of ALK-positive neuroblastoma.

## RESULTS

### Brigatinib inhibits ALK activity and abrogates proliferation of ALK addicted neuroblastoma cell lines

**Figure 1:**
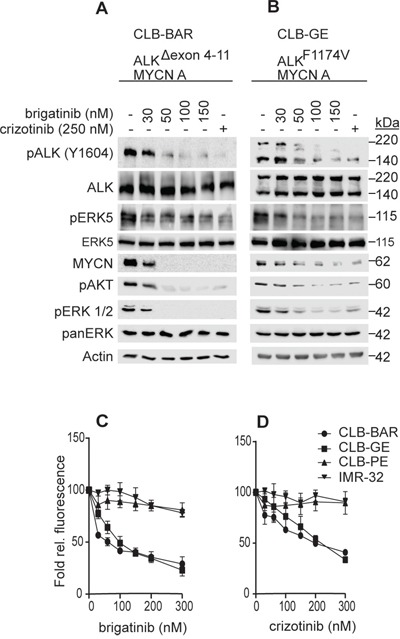
Brigatinib inhibits ALK activity and proliferation of ALK addicted neuroblastoma cell lines **A, B.** CLB-BAR (ALK-Δ4-11) and CLB-GE (ALK-F1174V), both ALK addicted cell lines, were treated with increasing doses of brigatinib for 6 hours. Crizotinib (250 nM) was employed as a positive control. Cells lysates were resolved on SDS/PAGE followed by immunoblotting for pALK (Y1604) and additional downstream targets as indicated. In the CLB-BAR cell line there is a genomic deletion in *ALK* between exon 4-11, resulting in an ALK band of approximately 170 kDa [21]. The CLB-GE cell line expresses a mutant full length version of ALK (F1174V) which is cleaved resulting in the detection of two bands with the antibody employed here. **C, D.** CLB-PE (ALK-WT) and IMR32 (ALK-WT) are ALK non-addicted neuroblastoma cell lines. Neuroblastoma cells were treated with increasing concentration of either brigatinib (C) and crizotinib (D) for 72 hours and cell viability was assessed by resazurin assay (Sigma, Sweden). Plotted values are means +/− SE from growth curves from at least three independent experiments performed in triplicate.

Brigatinib has been shown to inhibit ALK activity in NSCLC cell lines carrying the EML4-ALK fusion protein [6, 30]. In order to investigate the therapeutic efficacy of brigatinib in a neuroblastoma setting we employed several neuroblastoma cell lines, including CLB-BAR (*MYCN* amplification, *ALK* (Δ4-11) and amplified, ALK addicted), CLB-GE (*MYCN* amplification, ALK (F1174V) amplification, ALK addicted), IMR32 (*MYCN* amplification, WT *ALK*) and CLB-PE (*MYCN* amplified, WT *ALK*) [21, 31–33]. We have earlier shown that both CLB-BAR and CLB-GE cell lines are ALK addicted, while IMR32 and CLB-PE are not [34, 35]. ALK addicted cell lines were treated with either brigatinib or crizotinib, as a positive control for ALK inhibition, and their effect on ALK signaling analyzed by immunoblotting (Figure 1A, 1B). Brigatinib inhibited ALK phosphorylation in a dose-dependent manner, and at 50 nM brigatinib, phosphorylation of ALK was abolished (Figure 1A and 1B). Reduction of ALK phosphorylation by crizotinib was also observed, although at around 250 nM, in agreement with earlier published reports [7, 35]. In keeping with the inhibition of ALK phosphorylation, phosphorylation of downstream signaling targets such as ERK1/2, ERK5 and AKT were also affected and a decrease in MYCN levels was observed (Figure 1A, 1B).

While brigatinib and crizotinib both inhibited cell growth of ALK addicted neuroblastoma lines, they exhibited different IC_50_ values. The IC_50_ values observed for brigatinib and crizotinib in CLB-BAR were 75.27 ± 8.89 nM and 186.40 ± 17.28 nM, respectively, while in CLB-GE the IC_50_ values for brigatinib and crizotinib were 100.00 ± 17.53 nM and 225 ± 26, respectively (Figure 1C, 1D). Neither brigatinib nor crizotinib was able to inhibit growth of the non-ALK addicted neuroblastoma cell lines, IMR32 and CLB-PE, indicating that neither brigatinib nor crizotinib inhibitor was toxic to cells at the levels employed. Thus, brigatinib inhibits ALK activity in ALK addicted neuroblastoma cell lines more efficiently than crizotinib and displays a lack of toxicity at the cellular level.

### Brigatinib effectively inhibits constitutively active ALK mutants and neurite outgrowth

**Figure 2:**
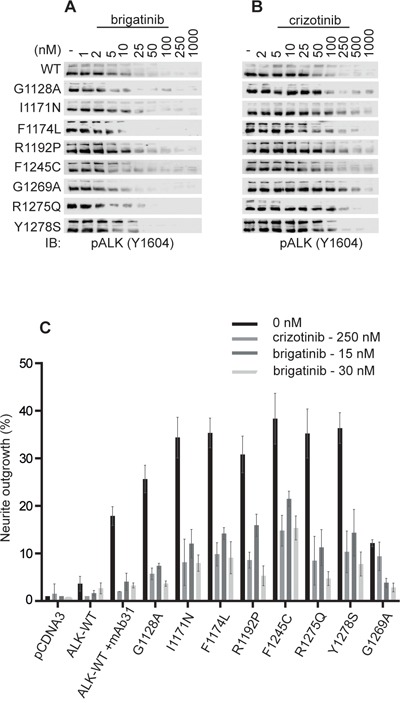
Brigatinib effectively blocks ALK activation and ALK-mediated neurite outgrowth in PC12 cells **A, B.** PC12 cells were transfected with ALK (0.75 μg). 48 hours post-transfection cells were treated with increasing amounts of either brigatinib or crizotinib. Wild type (WT) ALK was transfected with 1.5 μg pcDNA3-ALK-WT. Whole cell lysates were blotted for pALK (Y1604) to measure ALK activation. **C.** PC12 cells were co-transfected with pEGFPN1 together with the indicated ALK mutant. Wild type (WT) ALK was stimulated with 1 μg/ml of mAb31. GFP-positive PC12 cells were scored for neurite outgrowth after 48 hours treatment with either brigatinib or crizotinib (control) at the indicated concentrations.

We next examined the ability of brigatinib to abrogate the activity of mutated ALK variants found in neuroblastoma cases. Previously validated gain-of-function ALK mutants, such as ALK-G1128A, ALK-I1171N, ALK-F1174L, ALK-R1192P, ALK-F1245C, ALK-R1275Q and ALK-Y1278S were ectopically expressed in PC12 cells, in parallel with wildtype ALK, and inhibition of ALK phosphorylation by brigatinib or crizotinib was assessed by immunoblotting for pALK-Y1604 (Figure 2A, 2B). All loading ALK control blots for this experiment are shown as Supplementary Figure 1. We also included analysis of the ALK-G1269A mutant, which has so far not been observed as a neuroblastoma mutation but has been described as a more resistant mutation arising in EML4-ALK from NSCLC patients treated with crizotinib [36]. We observed that ALK-WT and ALK-F1174L were most sensitive to crizotinib whereas ALK-I1171N and the EML4-ALK secondary mutation mimic ALK-G1269A required relatively high doses of crizotinib for effective inhibition of ALK-Y1604 phosphorylation (Figure 2A, 2B, Table 1). Interestingly, brigatinib inhibited the activation of ALK gain-of-function alleles with a general IC_50_ of 5-35 fold less than that observed with crizotinib (Figure 2A, 2B, Table 1). These results suggest a superior inhibition profile for brigatinib as compared with crizotinib in a neuroblastoma setting. It is notable that ALK-I1171N and ALK-G1269A are more resistant to crizotinib with IC_50s_ of 111 and 119 nM, respectively. In contrast, brigatinib inhibits both the ALK-I1171N and the ALK-G1269A mutant receptors at low nM levels, 10 and 4, respectively.

**Table 1:** IC_50_ values for inhibition of ALK Y1604 phosphorylation by brigatinib or crizotinib in PC12 cells

ALK mutations	IC50 (nM)		Fold difference
	brigatinib	crizotinib	
WT	2.6± 0.2	19.68 ± 9	8
G1128A	2.0 ± 0.6	41.82 ± 5	21
I1171N	10.2 ± 5.7	110.90 ± 18	11
F1174L	1.5 ± 0.4	25.35 ± 10	17
R1192P	2.5 ± 0.4	64.0 ± 12	26
F1245C	6.6 ± 0.3	34.07 ± 0	5
G1269A	3.4 ± 2.0	118.9 ± 23	35
R1275Q	4.2 ± 0.7	32.58 ± 0	8
Y1278S	4.6 ± 0.2	72.44 ± 2	16

IC_50_ values were determined by quantification of Y1604 phosphorylation from the immunoblots in Figure 2A and 2B. Values represent mean ± SD from at least two independent experiments.

In agreement with the inhibition profiles observed for ALK-Y1604 phosphorylation, inhibition of ALK driven PC12 cell neurite outgrowth was also blocked by brigatinib. PC12s are a clonal rat adrenal pheochromocytoma cell line with enteric cell origin, which has the ability to differentiate and extend neurites upon extended ERK1/2 stimulation [37]. We and others have previously shown that activation of both human and mouse ALK triggers differentiation of PC12 cells into sympathetic-like neurons, a process that is characterized by extension of neurites [38]. This assay offers a convenient read out for ALK activity *in vitro*. Stimulated wild type ALK mediates neurite outgrowth which was abrogated by both crizotinib and brigatinib (Figure 2C). Further, brigatinib could abrogate neurite outgrowth of all tested ALK mutants. It should be noted that brigatinib, tested at both 15 and 30 nM blocks ALK-G1269A, ALK-R1275 and ALK-R1192P more efficiently than crizotinib (250 nM) (Figure 2C). Thus, brigatinib shows inhibition of multiple gain-of-function ALK neuroblastoma mutations including the “hot-spot” mutations as well as the crizotinib resistant ALK-G1269A mutation.

### Brigatinib blocks activity of the *ALK* gain-of-function alleles *F1174L* and *R1275Q in vivo*

**Figure 3:**
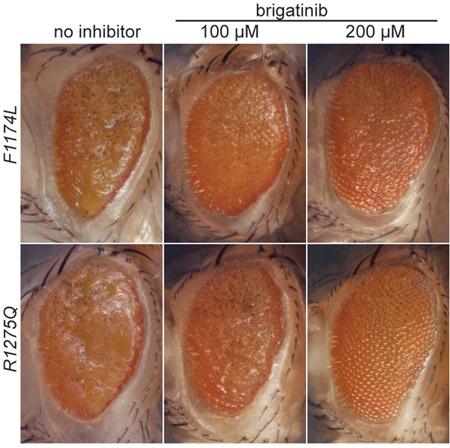
Effect of brigatinib on ALK gain-of-function rough eye phenotypes in a *Drosophila* ALK model Ectopic expression of gain-of-function “hot spot” ALK mutations ALK-F1174L and ALK-R1275Q in *Drosophila* eye imaginal discs with the *Gal4-UAS*-system results in a rough eye phenotype that is 100% penetrant [7, 38, 40]. Offspring that were grown on food containing brigatinib displayed a concentration dependent improvement of the rough eye phenotype.

To test if brigatinib can inhibit ALK gain-of-function mutations *in vivo*, we employed *Drosophila melanogaster as* a model organism by using the *Gal4-UAS*-system for tissue specific expression of the alleles *F1174L* and *R1275Q* in the eye imaginal disc [39]. *pGMR-Gal4* and *pGMR-Gal4*>*UAS*-*ALK* controls displayed a hexagonal arrangement of ommatidia similar to the wild type *w^1118^* and show that the ectopic expression of *pGMR-Gal4* or human ALK wild type does not lead to an eye phenotype (Supplementary Figure 2B, 2C). Transgenic flies expressing two gain-of-function variants of the human ALK receptors were generated to confirm their ligand-independent characteristics and also for testing of ALK inhibition as we have previously reported for NVP-TAE684 [7]. ALK gain-of-function “hot spot” mutations were ectopically expressed in the *Drosophila* eye disc, employing the *pGMR-Gal4* driver line, which directs protein expression in developing photoreceptors during larval stages (Figure 3). The expression of human ALK proteins was confirmed by immunostaining of eye discs using anti-human ALK antibody (Supplementary Figure 2A, 2B). Ectopic expression of the ALK gain-of-function alleles F1174L and R1275Q led to disrupted eye morphology in all offspring (100%), a so called ‘rough eye’ phenotype, which is characterized by disorganized ommatidia and missing interommatidial bristles, reflecting their robust ligand-independent activity (Figure 3) [38, 39, 41]. All larvae that were grown on brigatinib inhibitor-containing food developed into flies which showed a concentration-dependent improvement of the rough eye phenotype (Figure 3). Thus, brigatinib has the ability to abrogate rough eye phenotype in *Drosophila* ectopically expressing two different neuroblastoma “hot-spot” mutations.

### Brigatinib displays potent anti-tumor growth in a xenograft model of neuroblastoma

**Figure 4:**
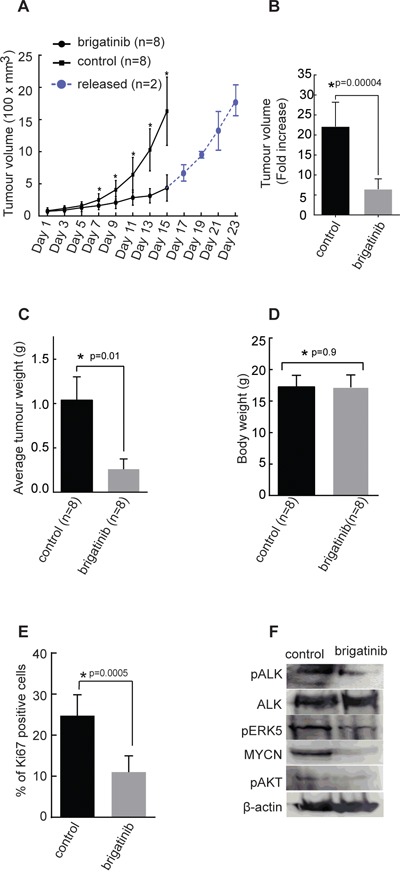
Effect of brigatinib in a xenograft neuroblastoma model 2.5×10^6^ CLB-BAR cells were injected into mice subcutaneously. In the first investigation mice (n=3) were employed and in the second investigation mice (n=5) were employed. **A.** Tumor growth curves represented as the average volume of either control group or brigatinib group (n=8 for each group, p=0.0002). In the second investigation where 5 mice were employed a subset of mice (n=2) were released from treatment after 14 days, for a further 8 days (blue curve). **B.** Average tumor volume in control and brigatinib treated groups after 14 days, p=0.00004. **C.** Average tumor weights in control and brigatinib treated groups after 14 days (p=0.01). **D.** Average body weight on the day at which mice were sacrificed after 14 days treatment with either brigatinib or vehicle control (p=0.9). **E.** Immunohistochemical staining of tumors with Ki-67 as a measure of proliferation rate as indicated, Ki-67-positive cells were counted manually per field of vision and quantitative results presented as mean ± SD (p=0.0005). Data are means +/− SE from 8 tumours, p values indicated; Student's *t* test. **F.** Immunonblotting analysis of indicated proteins from tumors collected after 8 days of treatment with vehicle or brigatinib.

We have earlier shown that crizotinib exhibits limited efficacy as a single agent treatment in orthotopic xenograft tumor growth and in the *TH-ALKF1174L/TH-MYCN* mouse model [42]. Therefore we examined the therapeutic effect of brigatinib as a single agent in BalbC/NUDE mice injected with human neuroblastoma CLB-BAR cells, (ALK Δ4-11 and MYCN amplified, ALK addicted) subcutaneously. Animals were treated for 14 days with brigatinib (50 mg/kg body weight, once daily via oral gavage) or vehicle control (Figure 4A). Examination of the tumor volume showed that brigatinib considerably inhibits proliferation of tumor growth *in vivo* (p=0.0004) (Figure 4A and 4B), and tumor weight was significantly reduced in the brigatinib treated group as compared with controls (p=0.01) (Figure 4C). Further, we observed an increase in tumor volume/growth when brigatinib treatment was discontinued after 14 days and for 8 days (n=2), indicating that tumor growth inhibition was due to brigatinib treatment (Figure 4A). The body weight of mice did not show any variation during treatment (Figure 4D) (p=0.9), and no obvious side effect of brigatinib treatment *in vivo* was observed. Analysis of excised tumor material with the Ki-67 proliferation marker indicated a significantly reduced proliferation in tumors from mice treated with brigatinib compared with controls (Figure 4E). Immunoblotting analysis of tumor material revealed decreased phospho-ALK levels in the brigatinib treated group, in line with our earlier findings (Figure 2). No obvious change in total ALK protein levels compared to control group were observed, however, MYCN, which is a downstream target of ALK activity showed decreased protein levels upon treatment with brigatinib (Figure 4F). Phosphorylated ERK5 and AKT, other downstream targets of ALK, exhibited reduced phosphorylation in the brigatinib treated group compared with the control group (Figure 4F). Therefore, brigatinib shows a robust and potent anti-tumor activity in this xenograft neuroblastoma mouse model.

## DISCUSSION

There has been a significant increase in targeted therapies available for the management of ALK-positive cancers including NSCLC, inflammatory myofibroblastic tumors (IMT), and anaplastic large cell lymphoma (ALCL). This improvement is due to the development of ALK inhibitors such as crizotinib, alectinib and ceritinib in the treatment of these ALK-related diseases [3, 42, 43]. Generally, treatment with ALK inhibitors shows an initial strong response in tumors harboring ALK fusion oncogenes, with a less encouraging response observed in ALK-positive neuroblastoma patients [16]. This low response in neuroblastoma patients may reflect the heterogeneity of the disease, and may also reflect the need for more potent ALK inhibitors in this tumor type. Neuroblastoma exhibits both numerical and segmental chromosome alterations [8, 17–20]. Further, *MYCN* and *ALK* are both located on the same chromosome, 2p, in close proximity to each other [3]. Therefore, amplification of *MYCN* can also involve *ALK* amplification, and neuroblastoma patients bearing both *MYCN* amplification and *ALK* mutations are characterized by an unfavorable aggressive neuroblastoma phenotype [26]. Finally, the recently reported ligands, FAM150A and FAM150B activate not only the wild type ALK receptor, but also super-activate constitutive active ALK neuroblastoma variants, offering additional options for the inhibition of ALK activity in NB [27].

Aberrant ALK activity occurs in about 5-7% of neuroblastoma cases but this percentage increases significantly in the relapsed patient population [8, 23, 24], thereby adding weight to the clinical relevance of ALK in neuroblastoma pathogenesis. The selection of intrinsic rare resistant clones by, and/or the emergence of acquired resistance to, crizotinib give credence to the need for second-generation ALK inhibitors such as brigatinib. Brigatinib, an ALK inhibitor, has been tested in crizotinib-refractory EML4-ALK-positive NSCLC patients, where about 72% of these patients showed encouraging responses [29, 44].

Treatment with brigatinib resulted in abrogation of cell growth and proliferation, as observed in this study. However, brigatinib inhibits ALK addicted neuroblastoma cell line proliferation with roughly 2 to 3-fold lower IC_50_ values compared with crizotinib. Previously described downstream targets of ALK such as ERK5 [35], AKT and ERK1/2 were clearly less phosphorylated upon treatment with brigatinib [7, 35]. MYCN amplification is associated with poor prognosis in NB patients, and there is evidence to the effect that MYCN cooperates with ALK in a synergistic manner to promote tumor growth [34]. Here we show that abrogation of ALK activity upon treatment with brigatinib also results in reduction of MYCN levels.

Multiple ALK point mutations have been identified in neuroblastoma, however, not all of them are classified as gain-of-function mutations, as some of the mutations are kinase dead and some seem to behave (at least in terms of activation) as wild type ALK [3]. It is clear that the constitutive active ALK mutations show differential sensitivity to ALK inhibitors such as crizotinib in both preclinical and also in clinical studies [45, 46]. Notably, ALK-G1269A, which is a secondary mutation found in a EML4-ALK-positive crizotinib-refractory NSCLC patients [36], together with the ALK-I1171N mutation, are more resistant to crizotinib. Brigatinib abrogated the activities of these mutant alleles with very low IC_50_ values. These observations indicate that the more resistant ALK-G1269A phenotype observed with crizotinib can be overcome by brigatinib. Brigatinib exhibits strong activity towards the most difficult to inhibit ALK neuroblastoma mutation among those investigated here (ALK-I1171N), inhibiting its phosphorylation with an observed IC_50_ value of 10 nM which is still low. The increased activity of brigatinib compared to crizotinib, is in the range of 5-35 fold based on our examination of the various constitutively active ALK mutations.

ALK mutations at residues F1174, F1245 and R1275 are often referred to as ‘hot spot’ mutations, together accounting for more than 80% of all sporadic ALK mutations found in neuroblastoma [14, 15, 26]. The robust rescue of the rough-eye phenotype caused by the ectopic expression of the hot-spot ALK gain-of-function mutations, ALK-F1174L and ALK-R1275Q, in *Drosophila* model system, confirms the ability of brigatinib to block aberrant ALK activity in an *in vivo* model system. Crizotinib is unable to rescue the rough-eye phenotype as observed with brigatinib [7]. In agreement, in an ALK/MYCN-driven mouse xenograft neuroblastoma model, brigatinib potently inhibited tumor growth, highlighting tumor inhibition efficacy *in vivo*. The abrogation of ALK phosphorylation in the excised tumor indicates that brigatinib does indeed block ALK activity within the tumor. Furthermore, the reduced levels of MYCN protein observed in tumors from mice receiving brigatinib are in keeping with our previous observations in cultured cells.

Brain metastases, particularly in non-small cell lung cancer patients, are among the common causes of disease progression with brain metastasis frequency in ALK inhibitor naive patients ranging from about 25 to 40%, which is high in patients with history of chemotherapy [47, 48]. However, in retrospective investigations of ALK-rearranged NSCLC patients treated with ALK inhibitors, mainly crizotinib, show a high incidence of CNS metastases, from about 45 to 70% [49–51]. Crizotinib has been reported to have poor penetration through the blood-brain barrier hence, the high incidence of brain metastasis in crizotinib refractory patients [52]. In a recent clinical trial of brigatinib in ALK-positive NSCLC patients, a radiological review of patients with baseline CNS metastases showed response in 50% (6/12) of patients, while in another subgroup of patients, 31% (8/26) with non-measureable CNS lesions had disappearance of all lesions [29], indicating that brigatinib is able to penetrate the blood-brain barrier. The overall incidence of brain metastasis in neuroblastoma patients ranges from 1.7 to 11.7% [53], making brigatinib a putative better treatment option than crizotinib.

In summary, we show that brigatinib abrogates ALK activation in neuroblastoma cell lines, *in vitro* biochemical assays as well as exogenously expressed ALK activity in flies and mice xenografts. Taken together, our data indicate that brigatinib is a potent inhibitor against ALK, supporting further exploration of brigatinib in an ALK-positive neuroblastoma setting.

## MATERIALS AND METHODS

### Generation of human ALK mutants

ALK mutants employed in this study were created in pcDNA3 by Eurofins MWG/operon (Ebersberg, Germany) as described by Chand *et al.* [38].

### Cell lines, antibodies and inhibitors

Neuroblastoma cell lines used were CLB-BAR (amplified MYCN/ALK, Δ4-11), CLB-GE (amplified MYCN/ALK, ALK-F1174V), CLB-PE (amplified MYCN, WT ALK) and IMR-32 (amplified MYCN, WT ALK) and PC12 cells were cultured and grown as previously reported [7, 35]. Primary antibodies employed for immunoblotting were: phospho-ALK (Y1604), phospho-AKT (S473), MYCN, phospho-ERK1/2 (1:2000), phospho-ERK5 (1:1000), ERK5, panERK, Actin (1:5000) from Cell Signaling Technology (Danvers, MA). Monoclonal antibody 135 (anti-ALK) was produced in the Hallberg laboratory against the extracellular domain of ALK [38]. Horseradish peroxidase conjugated secondary antibodies; goat anti-rabbit IgG and goat anti-mouse, IgG (1:5000) were obtained from Thermo Scientific. Brigatinib, was obtained from Ariad Pharmaceuticals and crizotinib was from Haoyuan Chemexpress Co., Limited, Shanghai.

### Cell culture, transfection, lysis, and immunoblotting

Neuroblastoma cell lines were cultured in RPMI-1640 medium supplemented with 10% fetal bovine serum (FBS) at 37°C, 5% CO_2_, 95% humidity. ALK addicted neuroblastoma cell lines, CLB-BAR and CLB-GE, were seeded into 6-well plates (0.5 × 10^6^ cells/well). Cells were treated with varying concentrations of brigatinib (from 0 to 150 nM) and with 250 nM crizotinib, in starvation medium (RPMI-1640 without FBS), for 6 hours. Cells were washed with cold 1X PBS and then harvested in lysis buffer (25 mM of Tris-Cl, pH 7.5, 150 mM of NaCl, 1% (v/v) Triton X-100, 1 mM DTT, protease inhibitor cocktail tablet (Roche)). Cell lysates were cleared by centrifugation at 14,000 rpm for 15 minutes at 4°C, after which the samples were boiled in SDS sample buffer, and subsequently analyzed by immunoblotting.

PC12 cells (3 × 10^6^) were transfected by electroporation in an Amaxa electroporator using 0.75 μg of ALK constructs (with the exception of 1.5 μg for WT ALK) and 100 μL of Ingenio electroporation solution (Mirrus Bio LCC). Cells from four electroporations were pooled together, mixed and equally seeded into 20 wells of 24-well plate with serial dilutions of the indicated inhibitors, for 4 hours. Cells were washed with cold 1X PBS and lysed with 1X SDS sample buffer (prepared from 4X SDS sample buffer - 3% SDS, 100mM Tris (pH6.8) 100 mM DTT, 50 mM EDTA, 40% glycerol). Samples were boiled at 95°C for 5 minutes and analyzed by immunoblotting. Phospho-ALK (Y1604) antibody was used to detect ALK phosphorylation. Intensity of pALK (Y 1604) and total (ALK) bands were quantified with Image Studio Lite 3.1. Data were normalized to the 0 nM inhibitor samples. GraphPad Prism 6.0 was used to calculate IC_50_ values by fitting data to a log (inhibitor concentration) versus normalized response (variable slope) equation.

### Proliferation assay

Cells were seeded at 15,000 per well with serial dilutions of the indicated inhibitors. After 72 hours cell viability was assessed by resazurin (Sigma, Sweden). IC_50_ values were calculated with GraphPad Prism 6.0 by fitting data to a log (inhibitor concentration) vs. normalized response (variable slope) equation. Each experiment was performed in duplicate and repeated at least three times.

### Neurite outgrowth assay

PC12 cells (2 x10^6^) co-transfected, in 100 μl of Ingenio electroporation solution (Minus Bio LCC), with pEGFPN1 (Clonetech) (0.5 ng) and ALK-mutant (0.75 ng) or ALK-wt (0.75 ng) as indicated were seeded sparingly into 24-well plates [38]. ALK-wild type was stimulated with 1 μg/ml mAb31. After 48 hours post transfection, the fraction of GFP-positive and neurite carrying cells versus GFP-positive cells were analyzed under a Zeiss Axiovert 40 CFL microscope. A cell was considered as neurite-carrying if it had neurite(s) that reached at least twice the length of the diameter of a normal cell body. The experiments were performed at least three times in triplicates.

### *Drosophila* transgenic lines and brigatinib treatment

The following *Drosophila* stocks from Bloomington Drosophila Stock Center (Indiana University) were employed: *w^1118^* and *pGMR-Gal4* (stock numbers 5905 and 9146, respectively). Generation of the *Drosophila* transgenic *ALK* lines was as described previously [7]. Transgenic *Drosophila* lines carrying the *UAS-ALK^F1174L^* or *UAS-ALK^R1275Q^* gain-of-function mutations were crossed with the *pGMR-Gal4* transgenic driver line to drive expression of the ALK mutations ectopically in the eye imaginal discs [38, 40]. Progeny of this cross were transferred at first instar larvae stage to food containing either 100 μM or 200 μM brigatinib (stock solution 10 mM in molecular grade ethanol dissolved at 25°C) in 2% molecular grade ethanol. Controls were grown in food containing 2% molecular grade ethanol. Over 100 larvae were included in this experiment, which was conducted at 25°C. Adult flies were collected and frozen at −25°C until microscopic analysis.

### Immunostaining of *Drosophila* eye imaginal discs

Imaginal discs from third instar larvae were fixed for 20 min in phosphate buffered saline (PBS) containing 4% formaldehyde. After 3 washes in PBS, discs were permeabilized for 10 min in PBS containing 1% Triton-X-100 and subsequently blocked for 1 hour in PBT (PBS containing 0.5% Triton-X-100 and 4% fetal bovine serum) at room temperature. Samples were incubated with anti-ALK antibody (monoclonal rabbit anti-ALK (D5F3; Cell Signaling #3633BC, dilution 1:200) in PBT overnight. After three washes in PBS secondary antibody (Alexa Flour A488, Jackson Immuno Research #111-545-144, dilution 1:1000) was applied in PBT for 2 h at room temperature. After three washes in PBS, discs were mounted in Flouromount G.

### Xenograft neuroblastoma model *in vivo*

Female Balbc/nude mice (Charles River, Germany) at 5-6 weeks age were maintained in a pathogen-free environment. For xenografts, 2.5 million CLB-BAR cells (MYCN amplified, ALK (Δ4-11) amplified) were injected subcutaneously. Once tumor volume achieved about 50mm^3^, drug was given at 50 mg/kg body weight, by oral gavage, once daily, continuously for 14 days. Mice were grouped by tumor volume randomly. Tumor volume was calculated with the following equation: V=0.5236×L×W^2^ (V=volume, L=longest, W=width). Brigatinib was formulated in 90% polyethylene glycol and 10% 1-methyl-2-pyrrolidinone solution.

### Immunohistochemistry

Mice were sacrificed and tumors were collected. Tumors were fixed in 4% PFA, followed by 15% and 30% sucrose in PBS. Tumors were embedded in OCT compound (VWR chemicals) and frozen at −80°C. For immunohistochemistry, samples were sectioned at 10 μm, blocked with 10% non-fat milk before incubating with primary antibody to detect Ki-67 (Cell Signaling Tech., Danver, MA) overnight at 4°C. Secondary antibodies (Cell Signaling Tech., Danver, MA) were applied for 2 hours at room temperature. Impact DAB (Vector Laboratories, SK 4105) was employed for staining followed by hematoxylin cross-staining (Sigma-Aldrich). After mounting sections, images were taken using an Axio zoom V16 stereo microscope (Zeiss).

### Ethical permission

All *in vivo* experiments were approved by Swedish legal regulations with the permission of ethical committees in Gothenburg (230-2014).

## SUPPLEMENTARY FIGURES


