# Supplementary material for: Brigatinib, an anaplastic lymphoma kinase inhibitor, abrogates activity and growth in ALK-positive neuroblastoma cells, Drosophila and mice
Source: Oncotarget. 2016 Mar 31;7(20):29011–22. doi: 10.18632/oncotarget.8508 (PMC5045374; doi:10.18632/oncotarget.8508)
Supplement: Supplementary file 1 [file oncotarget-07-29011-s001.pdf]

Brigatinib, an anaplastic lymphoma kinase inhibitor, abrogates activity and growth in ALK-positive neuroblastoma cells, *Drosophila* and mice

SUPPLEMENTARY FIGURES

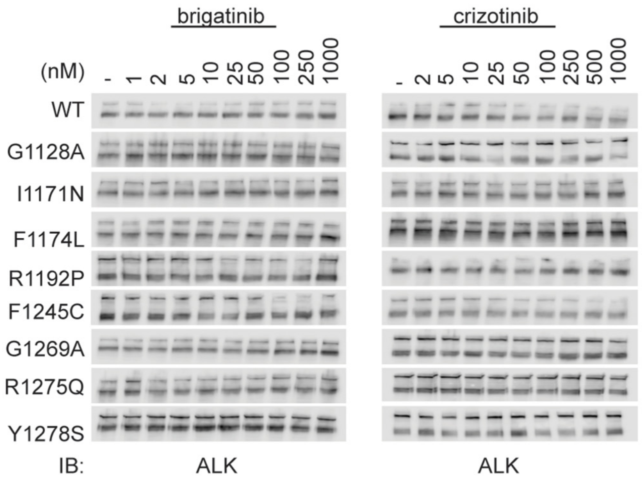

Supplementary Figure S1: Western blot analysis of ALK expression, in PC12 cell line, for all the indicated mutant ALK alleles, treated with either brigatinib or crizotinib.

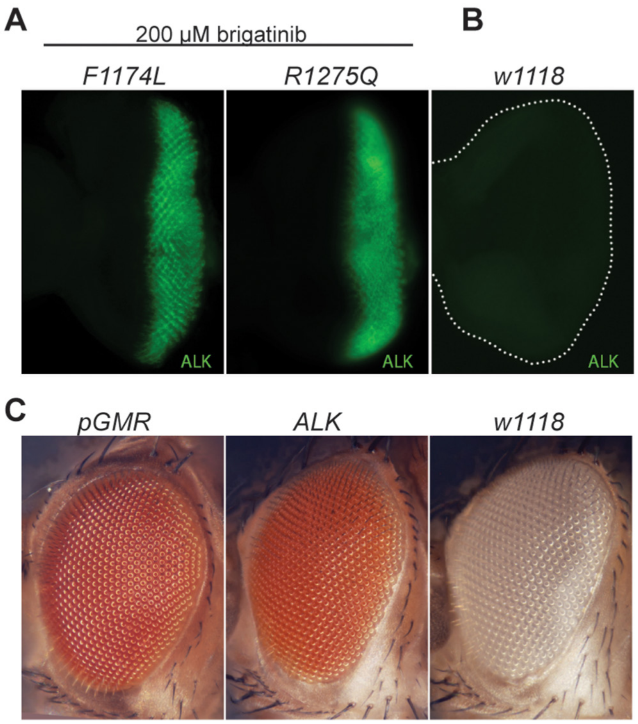

Supplementary Figure S2: Ectopic expression of human ALK mutations in Drosophila eye. A. Eyes discs of either ectopically expressed gain-of-function mutations ALK-F1174L or ALK-R1275Q in Drosphila developed and grown in 200 μM brigatinib. **B.** Control stain of w1118 drosophila eye. **C.** Eye of pGMR-GAL4 driver fly strain, followed by ectopic expression of wild-type human ALK in the adult eye which does not disrupt the wildtype organized pattern of ommatidia, indicating that the receptor is inactive. Finally, w1118, wild-type Drosophila eye displaying a highly organized pattern of ommatidia.
